# Supplementary material for: The Distinct Role of the HDL Receptor SR-BI in Cholesterol Homeostasis of Human Placental Arterial and Venous Endothelial Cells
Source: Int J Mol Sci. 2022 May 11;23(10):5364. doi: 10.3390/ijms23105364 (PMC9141204; doi:10.3390/ijms23105364)
Supplement: Supplementary file 1 [file ijms-23-05364-s001.zip › ijms-1600049-supplementary.pdf]

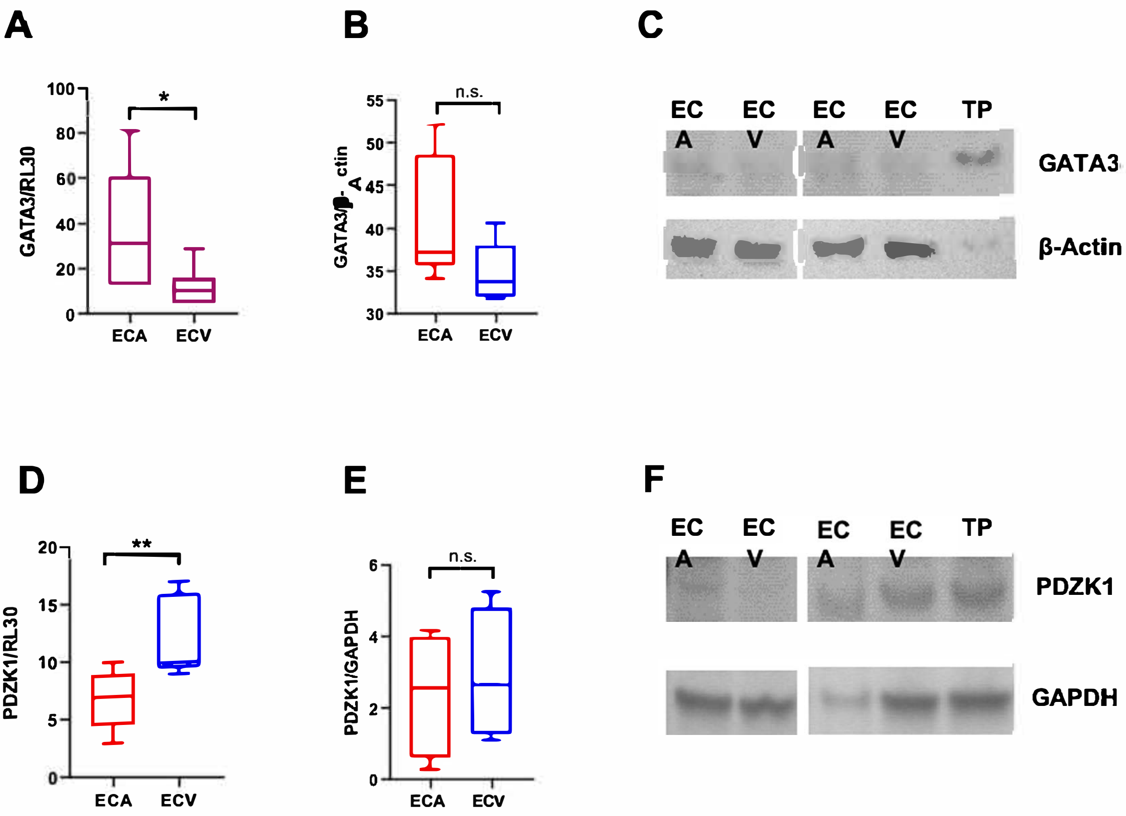

Figure S1: (A) RT-qPCR of GATA3 transcription factor in ECA and ECV showed more GATA3 mRNA in ECA than ECV (n=6, paired, Wilcoxon test). (B) The same observation, though insignificant, was made on protein level (Western blot, densitometric analysis; n=6, paired, Mann-Withney test). (C) Representative Western Blot against GATA3 in ECA and ECV. β-Actin was used as loading control for normalization. (D) RT-qPCR of PDZK1 demonstrated higher PDZK1 levels in ECV than ECA (n=5; Mann Whitney test). (E) On protein level (densitometric analysis of Western Blot), similar levels of PDZK1 in ECA and ECV were found (n=5, t-test). (F) Representative Western Blot against PDZK1, GAPDH was used as loading control for normalization.

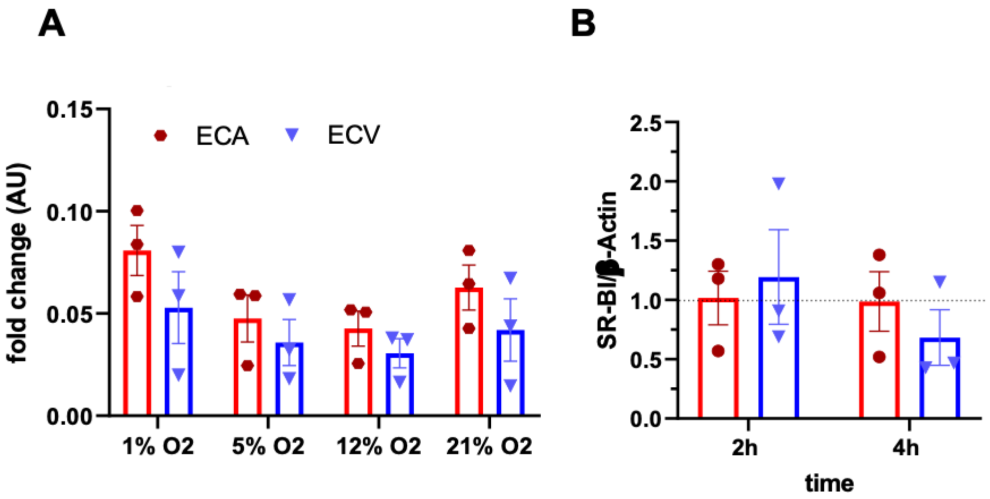

Figure S2: (A) RT-qPCR for SR-BI mRNA expression levels upon stimulation under different oxygen tensions. Hypoxia (1, 5, 12% oxygen, respectively) did not regulate SR-BI expression in either ECA or ECV compared to 21% oxygen tension. SR-BI expression in ECA was persistently higher than in ECV, though not significant. (B) Western blot densitometric quantification of the influence of shear stress on SR-BI protein in ECA and ECV. ECA and ECV were exposed to an appropriate degree of shear stress in relation to their respective vascular beds, for either 2h or 4h on an orbital shaker. The dashed line represents ECA and ECV not exposed to shear stress. ECA were unresponsive to shear stress, while SR-BI expression in ECV dropped by 40% after 4h, though not significant. n=3 different ECA/ECV.
